# Cardiovascular Diseases Among Indian Older Adults: A Comprehensive Review

**DOI:** 10.1155/2024/6894693

**Published:** 2024-06-25

**Authors:** Bisma Jan, Mohammad Imran Dar, Bharti Choudhary, Parakh Basist, Rahmuddin Khan, Abdulsalam Alhalmi

**Affiliations:** ^1^ Department of Biotechnology IILM University, Greater Noida, Uttar Pradesh, India; ^2^ Department of Cardiothoracic and Vascular Surgery All India Institute of Medical Sciences, New Delhi, India; ^3^ School of Medical and Allied Sciences K.R. Mangalam University, Gurugram, India; ^4^ Department of Pharmaceutics School of Pharmaceutical Education & Research Jamia Hamdard, New Delhi, India; ^5^ Department of Pharmaceutics College of Pharmacy University of Aden, Aden, Yemen

**Keywords:** burden, cardiovascular diseases, India, older adults, risk factors, WHO

## Abstract

Cardiovascular diseases (CVDs) constitute an important cause of morbidity and mortality globally, and India is no exception to this trend. With the ongoing aging of the population in India, there is a notable surge in the prevalence and impact of CVDs among older adults. This review is aimed at providing a comprehensive overview of the current knowledge concerning the prevalence, risk factors, and management of CVDs in the context of Indian older adults. The incidence of CVDs in India is not only alarming but also exhibits an upward trajectory with advancing age. Primary risk factors contributing to the elevated incidence among older adults include hypertension (HT), diabetes, dyslipidemia, obesity, smoking, a sedentary lifestyle, and poor dietary habits. Additionally, stress and genetic predisposition emerge as noteworthy contributors to CVDs in this population. Effectively identifying and managing these risk factors among older adults in India is imperative to alleviate the burden of these diseases and enhance overall quality of life. Strategies aimed at mitigating the impact of CVDs in the country necessitate a comprehensive approach, integrating lifestyle interventions, public health initiatives, and a robust healthcare system. In summary, CVDs represent a significant health concern in both rural and urban areas of India. However, variations exist in the prevalence, risk factors, and accessibility to healthcare between these regions. Therefore, addressing the prevalence of CVDs in India necessitates a complex, multidimensional strategy that takes into account the unique opportunities and challenges that come with living in both rural and urban areas.

## 1. Introduction

Cardiovascular diseases (CVDs) continue to be a significant hurdle, resulting in a higher number of deaths globally [1]. Compared to Europeans, CVDs affect Indians at least 10 years earlier and impact them during the most productive midlife period [2]. The noncommunicable diseases (NCDs) commonly include CVDs, various cancers, chronic respiratory illnesses, and diabetes, which are estimated to account for around 60% of all deaths [3]. In India, CVDs are the leading cause of death and disability and accounted for 31.8% of all deaths and 14.7% of disability-adjusted life years (DALYs) globally in the year 2017 [4]. Although India is experiencing a relative decline in the burden of CVD over the last few years as seen in many other parts of the world, it still remains the leading cause of death and DALYs in the country. This shift is posing a major challenge to public health in India [5]. The World Health Organization (WHO) estimates that in 2019, approximately 17.9 million individuals succumbed to CVDs, constituting 32% of all deaths worldwide [6]. In India, key risk factors for CVDs include high cholesterol, hypertension (HT), obesity, poor diet, diabetes, and sedentary lifestyle [7].

Heart attacks and strokes account for more than 80%, and over 17 million annual deaths are caused by CVDs. At present, India bears the greatest burden when it comes to acute coronary syndrome (ACS) and myocardial infarction, representing a 138% increase from 1990 [3, 8]. The prevalence of CVDs in India surpasses the global average by a significant margin. For instance, India's age-standardized death rate for CVDs (282 deaths per 100,000, with a range of 264–293) exceeds the global figure (233 deaths per 100,000, with a range of 229–236) [9]. One of the earlier conducted studies reported that the total number of persons affected by CVDs had nearly doubled, rising from 271 million in 1990 to 523 million in 2019 [10]. As per a study conducted by the World Economic Forum and Harvard School of Public Health, India is projected to incur economic losses amounting to around $2.17 trillion due to CVDs between 2012 and 2030 [11].

According to the Indian Census, 2011, the number of people aged 60 years and above had reached nearly 104 million, accounting for 8.6% of the nation's total population [12]. The aging process has a significant impact on the decline of cardiovascular function, which leads to a higher likelihood of developing CVDs among older adults [13]. Older adults in India have a higher risk of the dual burden of both communicable and NCDs, owing to their peculiar socioeconomic and cultural characteristics [14]. As per a study, almost one-third of the deaths occur in individuals who are under the age of 70, indicating a significant impact on premature mortality [15]. Furthermore, India is experiencing an increase in the elderly population due to demographic transition. These include inadequate nutrition, limited access to quality healthcare facilities, and a high prevalence of out-of-pocket healthcare expenses, which contribute to their increased health risks compared to other age groups [16]. The environmental shift is the most recent change in India that needs attention. The deteriorating ambient air quality in many parts of the country combined with persistent exposure to household air pollutants has emerged as a recent risk factor for the rising prevalence of CVD burden in India [17].

The diagnosis of heart failure in long-term care residents presents challenges because of the presence of multiple health conditions, cognitive impairments, extensive use of multiple medications, restricted mobility, and limited availability of treatments, particularly among older individuals [18]. Previously, a study utilized data from the Longitudinal Ageing Study of India (LASI) (2017–18), comprising 59,854 middle-aged and older adults. The findings of the study revealed higher proportions of heart disease diagnoses in older males (4.1%) and females (3.5%), with angina affecting 4.6% of males and 7% of females. Risk factors such as HT, diabetes, high cholesterol, and family history were associated with an increased likelihood of heart disease and angina, highlighting significant public health challenges among middle-aged and older Indians. The study concluded that individuals aged 70 years and above exhibited a greater prevalence of uncontrolled heart disease compared to middle-aged adults and younger older adults [19].

The exploration of CVD risk factors among older adults in India remains relatively limited. Consequently, it becomes crucial to conduct insightful evaluations of CVDs and their correlation with different other factors. Therefore, a comprehensive understanding of the various risk factors associated with CVDs, while emphasizing those that are pertinent to this age group, is imperative in order to develop an effective plan for managing and preventing CVDs in older adults in India. Henceforth, this study is aimed at analyzing the burden and prevalence of CVDs together with associated risk factors among the elderly population of India. It also depicts strategies to improve the CVD prevention and discusses various policies for Indian citizens. Furthermore, it enlists the availability of several essential medicines of CVDs in India accompanied by a range of diagnostic techniques. This compilation is a step forward in addressing several issues pertaining to CVDs in the aging population of India and calls for the intervention of healthcare professionals, policymakers, and regulators towards the effective management of the problem.

## 2. Methodology

### 2.1. Literature Search Strategy

A systematic literature search was conducted in major electronic databases including Medline, PubMed, Web of Science, and Google Scholar, as well as the websites of the WHO, Indian Council of Medical Research (ICMR), and other relevant sources. The search was focused on identifying articles published in English from the year 2006 onwards, with the aim of capturing the most recent literature on CVDs among older adults in India.

### 2.2. Inclusion Criteria

Articles reporting on CVDs among older adults in India were included. No restrictions were applied based on study design, community type (rural/urban), or gender. Studies conducted in any setting (community-based, facility-based, or workplace-based) were considered. Only studies reported as full text were included in the review.

### 2.3. Search Terms

Key search terms used included “cardiovascular diseases,” “older adults,” “risk factors,” “obesity,” “diabetes,” “hypertension,” “burden,” and “India.” These terms were selected to ensure the comprehensive retrieval of relevant literature pertaining to the prevalence, risk factors, and burden of CVDs among older adults in the Indian context.

### 2.4. Exclusion Criteria

Unpublished data were excluded from the review to maintain the reliability and quality of the included studies.

### 2.5. Data Collection and Selection Process

After the initial literature search, duplicates were removed, and the remaining articles were screened based on title and abstract to assess their relevance to the topic. Full-text screening was then performed to further evaluate the eligibility of studies for inclusion in the review. Data from the LASI were also utilized to supplement information on CVDs among older adults.

### 2.6. Data Analysis

Selected studies were subjected to a detailed review, with a focus on their relevance to the topic of interest. Data extraction was performed to capture key findings related to the prevalence, risk factors, and burden of CVDs among older adults in India. The extracted data were synthesized to provide a comprehensive overview of the current state of knowledge in this field.

### 2.7. Final Selection

Following the review process, a total of 121 references were selected for inclusion in the review article. These references predominantly focused on CVDs among older adults in India and provided valuable insights into the epidemiology and determinants of CVDs in this population.

### 2.8. Quality Assessment

Quality assessment of the included studies was not explicitly mentioned in the methodology, but the inclusion of studies published in reputable peer-reviewed journals and reports from reputable organizations contributed to the overall reliability of the findings.

## 3. Burden of CVDs in India

CVD is a significant health burden in India. This trend is expected to persist in the coming years, creating a considerable economic and social burden on the Indian population [20]. According to the Global Burden of Disease study age-standardized estimates (2010), nearly a quarter (24.8%) of all deaths in India are attributable to CVDs [21]. Factors such as sedentary lifestyles, unhealthy diets, tobacco use, and rising rates of obesity are contributing to the increasing prevalence of CVDs in the country. The increase in CVDs in India can also be attributed to a change in diet that is characterized by a decrease in the intake of nutrient-dense foods like whole grains, legumes, fruits, and vegetables and an increase in the intake of meat products, processed foods, foods high in salt, and high energy ready-to-eat foods [22].

Further, socioeconomic and cultural factors are also contributing to burden of the diseases in India. Individuals with lower socioeconomic status are less inclined to receive screenings for blood pressure (BP), glucose, and cholesterol and have limited knowledge of cardiovascular risk factors (CVRFs). India has also profound and long-standing social and economic disparities, resulting in a large portion of the population lacking access to affordable healthcare [23]. In India, around 38% of elderly individuals are hospitalized because of communicable diseases, 52% due to NCDs, and 10% due to injuries and other causes [24]. Social determinants of health are a multidimensional measure encompassing factors related to where people are born, raised, engage in activities, reside, and work, which exert a significant influence on health outcomes, including HT and CVD [25].

Furthermore, in India, individuals in lower socioeconomic positions (SEPs) tend to consume less fruit and engage in higher rates of tobacco and alcohol use compared to those in higher SEP. Conversely, higher SEP individuals exhibit greater instances of overweight, physical inactivity, diabetes, HT, and family history of CVDs (specifically in men). Recently, a study was carried out using a cross-sectional design, in villages within each of rural Trivandrum, West Godavari, and Rishi Valley. Sampling was stratified by age group and sex. Trivandrum, the region with the highest SEP, had the greatest prevalence of HT, whereas Rishi Valley, the lowest SEP region, had the least. In interaction analyses, there was no evidence that educational attainment modified the association between income and HT [26]. Analysis of the 75th round of the National Sample Survey in 2018 reveals significant differences between Empowered Action Group (EAG) and non-EAG states in terms of economic dependency, chronic illness prevalence, and insurance coverage among the elderly in India. Elderly individuals in non-EAG states face twice the risk of chronic illness and are more likely to have insurance. Factors such as gender, caste, education, and income play significant roles in shaping these disparities, emphasizing the need for targeted policies to address healthcare access and economic security for the elderly population [27].

Several case-control studies in India have confirmed about the factors that contribute significantly to the development of CVDs, including but not limited to tobacco usage, obesity, high BP, high LDL (low-density lipoprotein), low HDL (high-density lipoprotein) diabetes, inadequate intake of fruits and vegetables, and sedentary lifestyle [22]. According to recent studies, CVDs account for nearly one-third of all deaths in the country, with a prevalence rate of around 7.5%. The burden of CVDs in India is expected to increase in the coming years due to the aging population, changing lifestyles, and rising rates of obesity, diabetes, and HT [23]. Studies have shown that more than 80% of deaths and 85% of disability related to CVDs occur in low- and middle-income countries (LMICs), including India. CVDs affect Indians more frequently and at a younger age than individuals in developed countries [24].

The global burden of CVDs has risen significantly, particularly in LMICs like India. This is primarily due to factors such as population growth and aging. As a result, the burden of CVDs has shifted considerably towards LMICs [25]. India has established regular and structured surveys to examine the health of its population. These surveys are conducted in selected states and provide reliable and comprehensive data on CVD risk factors in the population [28]. Surveillance in India is not well integrated, and there are gaps in the health management information system at the national level. This system has not been given sufficient attention until recently [29]. India's per capita health expenditure in the year 2014 was calculated to be $253, accounting for 4.5% of India's gross domestic product (GDP). Only a fraction of India's total health expenditure, amounting to 31.3% or 1.1% of the GDP, is contributed by the Indian government [30]. India does not have a comprehensive national surveillance system that covers the entire population to estimate the prevalence of CVDs and their trends over time. Nonetheless, recent findings from three major longitudinal studies in India indicate that a considerable proportion of deaths, ranging from 30% to 42%, can be attributed to CVDs. The studies also reveal that age-standardized mortality rates due to CVDs range from 255 to 525 per 100,000 in men and 225 to 299 per 100,000 in women [21]. Recently, a study confirmed that in India, CVDs account for 28.1% of all deaths and 14.1% of all DALYs in 2016, a significant increase from 15.2% and 6.9%, respectively, in 1990 [31]. Another study reported that the years of life lost attributable to CVDs in India have increased by 59% from 1990 to 2010 (23.2 million to 37 million) [21].

CVDs, being a substantial health burden, contribute to a high number of deaths and disabilities across the population in India. Addressing this issue requires comprehensive public health interventions, including promoting awareness about risk factors, encouraging healthy lifestyles, improving access to healthcare services, and implementing effective preventive measures. Failure to address the burden of CVDs could have profound implications for the health and well-being of the Indian population and strain healthcare systems [21].

### 3.1. Prevalence of CVDs Among Older Adults in India

In India, there is a high prevalence of CVDs, especially in older adults. In 2016, the predictable prevalence of CVD in older adults in India was estimated to be 54.5 million [20]. As of 2016, all states in India have an epidemiological transition ratio of less than 0.75. Indians experience the onset of CVD approximately 10 years earlier than individuals in European countries, affecting them during their most productive midlife period [31]. To illustrate, while only 23% of CVD-related deaths occur before the age of 70 in western populations, this figure rises to 52% in India. As per another study, India experienced a loss of around 62.5 million years of life before the expected lifespan due to CVD in the year 2016 [32]. One of the latest researches conducted revealed that 29.4% of adults aged 45 and above in India have a confirmed diagnosis of CVD based on self-reporting. Older age is a risk factor for CVD, and women are more susceptible than men. The prevalence of CVDs was found to be higher in rural areas than in urban areas as per a study [33].

Every year, India experiences over 10.5 million deaths, with 20.3% of male deaths and 16.9% of female deaths reportedly CVDs [34]. The occurrence of CVDs is notably higher among older individuals who are obese or overweight (56.7%), have a high-risk waist circumference (56.3%), and those with a high-risk waist–hip ratio (37.9%). Additionally, CVDs are more prevalent among older adults who do not engage in regular physical activity (38.8%). Female older adults reported a significantly higher prevalence of CVD compared to males (38.8% versus 31.1%) [35]. Over the last 30 years of the previous century, there was a notable rise in the prevalence of ischaemic heart disease in both rural and urban settings in India. Specifically, the prevalence of this disease raised from 2% to 5% in rural areas and from 7% to 11% in urban areas [36]. A study reported that the prevalence of CVD among adults with age 60 years and above in India was 33.6%. The same study also found that the prevalence of HT was 63.8% among older adults in India. According to self-reported data in the year 2017 (LASI (Wave 1, 2017–18) (*N* = 65562)) conducted on adults aged 45 years and above, almost 30% were diagnosed with CVD. The likelihood of being diagnosed with CVDs increases with age, from 22% in the 45–54 age group to 38% in those aged 70 or above. As per the study, women had a higher prevalence of diagnosed CVDs (32%) than men (26%), and those living in urban areas had a higher prevalence (40%) compared to those in rural areas (25%). The study further reported that older adults who have diabetes (66%) or high cholesterol (68%) are more likely to have been diagnosed with CVDs. On the other hand, there is a clear negative relationship between physical activity levels and the prevalence of CVDs. In other words, the more physically active an older adult is, the less likely they are to have been diagnosed with CVD [7]. Another study reported that in India, the prevalence of self-reported risk factors for CVDs among older adults was 21 out of 100 people. HT and diabetes were the most prevalent risk factors, accounting for 38% and 31%, respectively, of all reported risk factors [37]. The prevalence of CVD varies across different regions, not just in terms of overall numbers but also in specific areas. The prevalence of coronary heart disease (CHD) varies between different population settings. In rural areas, the reported occurrence of CHD ranges from 1.6% to 7.4%, while in urban areas, it ranges from 1% to 13%. According to reports, the prevalence rate of CHD in urban areas of certain northern states such as Delhi, Jammu, Kashmir, and Uttar Pradesh and western states such as Rajasthan is approximately 6%–10%. Meanwhile, the rural areas of Jammu and Kashmir have a prevalence rate of 6%–7%, whereas Himachal Pradesh and Punjab have a rate of 3%–5%. Rajasthan also has a rate of 3%–5%. In Andhra Pradesh, the overall prevalence of CVD was found to be 5.4% [38]. Migrant Asian Indians have a prevalence of coronary artery disease (CAD) that is three times greater than that of the native population. Compared to other ethnic groups, Indians have a higher likelihood of being hospitalized for complications related to CAD, with hospitalization rates ranging from two to four times more frequent [3].

The occurrence of common risk factors for CVDs, which can be listed as high BP, abnormal lipid levels, overweight, and diabetes, differs significantly across countries and has evolved over time. New risk factors for CVDs have been discovered and are receiving significant attention, as conventional risk factors cannot explain over 60% of CHD cases in native Indians. Studies comparing these newer risk factors have revealed that Indians tend to have higher levels of plasminogen activator inhibitor (PAI-1), C-reactive protein, and homocysteine [39]. Unhealthy diet, physical inactivity, tobacco use, and harmful alcohol consumption are the most common significant behavioral risk factors for heart disease and stroke among older adults in India [40]. These factors can lead to intermediate risks such as high BP, raised blood glucose, overweight, and high serum lipid levels, which shows an increased probability of stroke, heart failure, heart attack, and other cardiac-related complications. Various factors, such as globalization, urbanization, population aging, poverty, stress, and hereditary traits, also contribute to CVDs.

There are several other risk factors associated with CVDs among older adults in India. These include older age, people residing in rural areas, high cholesterol, diabetes, and being physically inactive. Several research studies have also evaluated the regional patterns of risk factors in India, which contribute to the escalating prevalence of CVDs (Table 1).

## 4. Risk Factors for CVDs

### 4.1. HT

**Figure 1:**
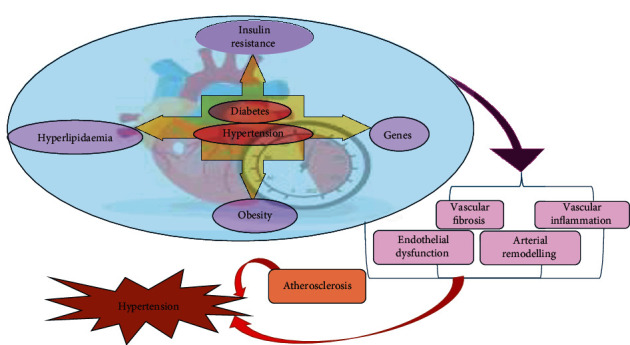
Common risk factors that contribute to the development of diabetes and hypertension.

CVD is the primary cause of death worldwide, and high BP is one of the most significant risk factors associated with it. This risk is present across all demographic groups [42]. High BP, also known as HT, is a prevalent NCD and a significant public health issue that accounts for 19% of all NCD-related deaths worldwide. People with metabolic disorders, such as insulin resistance, diabetes, and cardiometabolic syndrome, often exhibit a significant prevalence of HT, which is a strong contributor to the risk of CVDs (Figure 1) [43]. Commonly, elevated BP is accountable for around 54% of stroke cases and 47% of CHD occurrences. This medical condition is prevalent, and its incidence increases with age, affecting around 65% of individuals aged 60 years and above. Consequently, the impact of high BP on mortality among older adults is expected to rise in the coming years [44, 45]. BP-related diseases are becoming a growing problem in countries like India and China due to various factors, including an aging population, rising urbanization, and an increase in conditions like stroke among specific age groups. As a result, there is an increasing burden of these diseases on the healthcare system in the aforementioned countries [46].

According to community surveys conducted in different states of India, there has been a significant surge in the HT over the past three to six decades. In urban areas, the prevalence of HT has increased about 30 times, while in rural communities, it has increased about 10 times. The prevalence of HT is higher in urban areas than in rural areas [47]. One of the earlier conducted studies, among older adults, found that there is a high incidence of HT and other risk factors for CVDs in Central India. It suggests that public health measures are needed to address this trend in both communities, through educational and awareness-raising programs about lifestyle changes such as weight reduction, reduced tobacco use, increased physical activity, and healthier diets. It also highlighted the importance of regular screenings for BP, blood sugar, and cholesterol levels [48]. The prevalence of HT increases with age, and a higher percentage is reported in men in each age group, except for those aged 65 years and older where the percentage was almost the same between men and women. After adjusting for the 2011 census population survey of India, the prevalence of HT was 29.7%, while after adjusting for the WHO reference population, it was 32.8%. This study revealed a considerable incidence of HT among Indian older adults, with nearly one-third of the participants affected by the condition. Given that there are approximately 762 million Indian adults aged 18 and above, this suggests that there are currently around 234 million adults in India who have HT. In conclusion, the prevalence of HT was approximately 30% among individuals aged 18 years and above and 52% among those aged 65 years and above [49].

According to another recent study, the incidence of self-reported HT adjusted for age and sex was 25.8% in India, with significant variations observed among different states [50]. Another study concluded that two-thirds of older Indian adults had HT, with the majority being undiagnosed or diagnosed but not adequately controlled. As per this study, the total HT prevalence among older adults in India was 63.2% (41.5% diagnosed and 21.6% undiagnosed). Among those with HT, 34.5% were undiagnosed, 34.2% were diagnosed but uncontrolled, and 31.3% were diagnosed and controlled [51]. A meta-analysis of 142 community-based studies conducted in India showed notable variations in the occurrence of HT in different regions of India. The prevalence of HT in rural regions varied from 14.5% in the north to 31.7% in the east, while in urban regions, it ranged from 28.8% in the north to 35.8% in the west [47].

HT is a prevalent health concern among older adults in India, with a considerable portion of the population affected. About 33% of urban and 25% of rural Indians are hypertensive. Of these, 25% of rural and 42% of urban Indians are aware of their hypertensive status. Only 25% of rural and 38% of urban Indians are being treated for HT. One-tenth of rural and one-fifth of urban Indian hypertensive population have their BP under control [47]. Hence, the burden of HT among older adults in India underscores the importance of targeted preventive strategies, regular screenings, and effective management to reduce the risk of cardiovascular complications and improve overall health outcomes.

### 4.2. Obesity

Obesity affects more than 135 million individuals in India and is considered to be a major risk factor for developing several types of CVDs. Obesity is increasingly becoming a significant health concern in both developed and developing nations, closely associated with key CVRFs. The WHO recommends all individuals decrease consumption of total fat and saturated fat [52]. Prevalence rates of obesity in India differ depending on various factors including age, gender, place of residence, socioeconomic status, and criteria used for the measurement of obesity [53]. In Asian Indians, abdominal obesity has been recognized as an important risk factor for CVDs. Furthermore, with the increase in the elderly population, the risk of CVDs among Indian older adults is also increasing. In India, obesity has been considered to have reached a pandemic level globally and is found across all age groups [54].

Approximately 21% of individuals aged 45 and above in India's elderly population are overweight, while around 7% are classified as obese. Among this demographic, those residing in urban areas and belonging to higher socioeconomic groups exhibit a higher prevalence of obesity compared to their counterparts. These findings align with previous research and demonstrate a consistent trend in obesity prevalence among the elderly population in India [55]. In the multivariate-adjusted model, it was found that Indian adults aged 45 and above have a significantly increased number of developing CVDs and other NCDs when compared to their normal counterparts. Specifically, obesity was associated with a 2.3 times higher likelihood [56]. Hence, the burden of obesity along with physical inactivity increases the risk of CVDs in older adults and there is an urgent need for framing direct and indirect strategies to control obesity in order to reduce the burden of CVDs among older adults in India [52].

### 4.3. Dyslipidemia

Over the past two decades, there has been a scarcity of extensive studies focusing on the epidemiology of cholesterol and other lipoprotein lipids in large sample sizes in India [48]. The prevalence of various forms of lipid abnormalities among Indians has been reported only in a few studies (Table 2). The development of CHD is significantly influenced by lipid dysfunctions such as elevated levels of total cholesterol, LDL, very LDL (VLDL) cholesterol, and triglycerides (TGs), as well as decreased levels of HDL cholesterol (HDL-C). In particular, an increase in LDL cholesterol is strongly linked to the onset and advancement of CAD. Analysis of studies conducted on the Indian older adult population indicates a rise in the average levels of total cholesterol. According to recent studies, around 25%–30% of people living in urban areas and 15%–20% of those in rural areas have elevated cholesterol levels [57]. One significant limitation of epidemiological studies in India is the absence of comprehensive large-scale studies that provide in-depth information on patterns of dyslipidemia. In contrast to western populations, individuals of Indian and South Asian descents generally exhibit elevated TG levels and decreased levels of HDL, whereas total cholesterol levels tend to be lower than those found in the United States and the United Kingdom [58]. The India Heart Watch study involved measuring fasting lipid levels in urban middle-class subjects across 11 cities in India. After adjusting for age, the prevalence of different cholesterol and lipoprotein abnormalities was found to be as, for men and women, total cholesterol levels equal to or greater than 200 mg/dL in 25.1% and 24.9%, respectively, and LDL cholesterol higher than 130 mg/dL in 16.3% and 15.1%, respectively, and higher than 100 mg/dL in 49.5% and 49.7%, respectively. HDL-C levels were below 40 mg/dL in men and below 50 mg/dL in women in 33.6% and 52.8%, respectively. The total cholesterol ratio was equal to or greater than 4.5 in 29.4% and 16.8% of men and women, respectively. TG levels were equal to or greater than 150 mg/dL in 42.1% and 32.9% of men and women, respectively [59]. The Jaipur Heart Watch (JHW) is a set of cross-sectional studies conducted on a population residing in an urban area in India. These studies reported changes in cholesterol and other lipoproteins over a period of 20 years.

ICMR-India Diabetes (ICMR-INDIAB) study's first phase was carried out in one union territory and three states of India (Maharashtra, Jharkhand) and Tamil Nadu, and it included a representative population. The study found that among the participants, 13.9% had hypercholesterolemia, 29.5% had hypertriglyceridemia, 72.3% had low levels of HDL-C, 11.8% had raised levels of LDL-cholesterol, and 79% had anomalies in at least one of the lipid parameters [60]. According to the latest survey in Punjab, 27% of adults have high levels of cholesterol or TGs. Specifically, 9.8% have high cholesterol levels, and 21.6% have high TG levels, and this is true regardless of whether they live in urban or rural areas and whether they are male or female. The survey also found that having more than two medical conditions, as well as having diabetes and high BP, are linked to all types of dyslipidemia [61]. Therefore, the occurrence of dyslipidemia is alarmingly raised in India, which necessitates the implementation of immediate lifestyle intervention measures to prevent and control the critical CVRF.

### 4.4. Tobacco Smoking and CHD in Indian Older Adult Population

Tobacco use, including smoking, is a significant contributor to the development of CVDs, and this risk is present even with minimal exposure to cigarette smoking [62]. In India, tobacco usage is primarily done in two ways: through smoking and smokeless tobacco. The most commonly used forms of smokeless tobacco in India are tobacco mixed with lime and tobacco mixed with pan, tobacco pan masala, and betel quid. Smokeless tobacco consumption is prevalent in about 20% of the population in India, with a higher percentage of usage in males (28%) than females (12%) and in rural areas as compared to urban areas [63]. In India, men tend to smoke consistently throughout their lives, whereas women usually start smoking at a later age [64]. The relationship between tobacco smoking and morbidity and mortality caused by CHD is widely recognized. CHD is responsible for 16.6% or 9.4 million deaths annually worldwide, and smoking is the cause of 18% or 1.62 million deaths from CHD globally. Additionally, smoking leads to a significant loss of health due to CHD, estimated to be 40.6 million DALYs [65]. Tobacco consumption has significant social and economic implications, apart from the loss of human lives. In India, the total economic cost linked to tobacco usage in individuals aged 35 years and above for all diseases in the year 2017-18 was INR 177,341 crore (equivalent to USD 27.5 billion) [66]. Tobacco use, including both smoking and smokeless forms, as well as exposure to second-hand smoke, can lead to the development of heart disease through various pathways such as inflammation, constriction of blood vessels, formation of blood clots, and decreased oxygen supply [67]. Individuals who smoke have elevated levels of extracellular lipid content within their plaque, increasing the susceptibility to rupture [68].

In India, smoking is believed to be accountable for approximately one out of every 20 deaths among women and one out of every five deaths among men aged 30–69 years, with a significant number of deaths attributed to CVDs. According to a study conducted in India in 2013, a combination of smoke-free regulations and higher tobacco taxes could prevent 25% of heart strokes over 10 years [69]. Recently, it was observed that the consumption of smokeless tobacco is higher among male older adults than in female older adults; however, a higher percentage of female older adults consumed smoked tobacco than their male counterparts [70]. A more recent study in northeastern India found increased tobacco usage among middle-aged and older women, emphasizing targeted interventions for widowed, separated, or unmarried individuals; alcohol consumers; and those with lower socioeconomic status or education [71].

**Figure 2:**
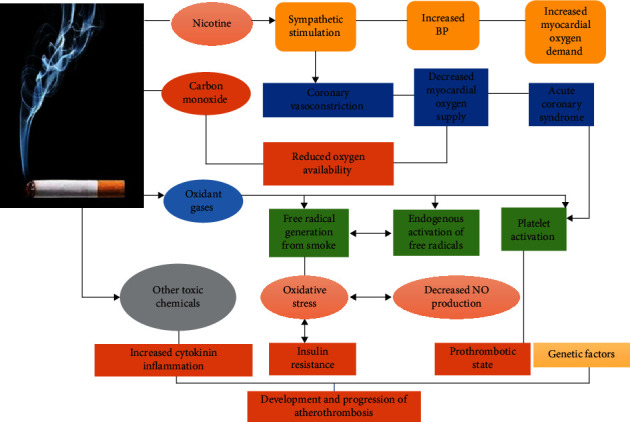
Pathophysiological mechanisms of tobacco in the progression of cardiovascular disease in India. BP, blood pressure; NO, nitric oxide.

Until 2016, India ranked as the world's second-largest tobacco consumer, following China closely. However, according to the Global Adult Tobacco Survey-2 report released in June 2017, there was a 6% decrease in the usage of tobacco among older adults in Indian states [72].  Figure 2 provides an overview of the pathophysiological mechanisms through which tobacco contributes to the development of CVDs.

### 4.5. Diabetes

It is estimated that in India, 77 million people suffer from diabetes; thus the country is considered the second highest diabetic capital in the world followed by China [73]. In 2013, there were 65.1 million individuals aged between 20 and 79 years afflicted with diabetes, with projections indicating an increase to 109 million by 2035 [74]. Individuals with diabetes have a higher chance of developing CAD, with two to four times greater risk than those without diabetes [75]. Therefore, the rise in the occurrence of diabetes can also imply an increase in the likelihood of developing CAD. Adults with diabetes have been found to have a greater likelihood of having CVDs compared to those without diabetes. The risk of CVDs also rises as blood glucose levels increase and is a required parameter for a diabetes diagnosis [76].

The prevalence of diabetes in the age group of 20–70 years in India is 8.8%, making it a significant challenge in the country [77]. The increase in NCDs like diabetes is attributed to various factors such as urbanization, globalization, sedentary lifestyles, obesity, tobacco usage, and unhealthy diets. One of the earlier conducted studies examined the geographical distribution of diabetes prevalence among the elderly population in India and found that southern India exhibits higher prevalence compared to the northern, eastern, and central regions of the country [78]. The prevalence of diabetes was most pronounced among older adults, particularly those aged 60 years and above, due to factors such as rapid urbanization, adoption of unhealthy western dietary habits, and lack of physical activity [79].

**Figure 3:**
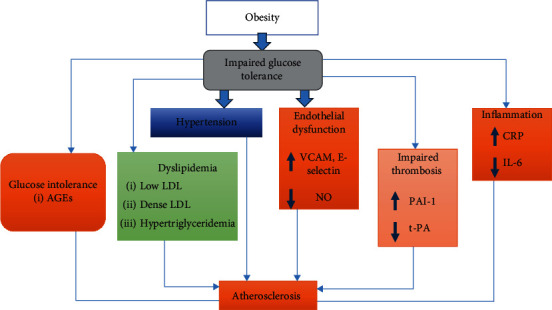
Correlation of insulin resistance with cardiovascular risk factors and atherosclerosis. AGEs, advanced glycation end products; CRP, C-reactive protein; IL-6, interleukin 6; LDL, low-density lipoprotein; NO, nitric oxide; tPA, tissue plasminogen activator; VCAM, vascular cell adhesion molecule.

Adopting healthy behaviors like consuming a balanced diet and engaging in regular physical activity can significantly decrease the burden of diabetes in India among older adults [3]. The abnormally raised glucose levels in diabetes destroy the function of blood vessels and nerves, leading to various complications such as atherosclerosis, HT, and dyslipidemia, all of which increase the risk of CVDs. Therefore, managing diabetes is crucial in minimizing the risk of developing CVDs and its associated complications [80]. The progression of obesity leads to the development of insulin resistance, which further increases the risk of developing CVDs as shown in Figure 3.

### 4.6. Physical Inactivity

Despite almost 50% of the Indian population being vegetarian, the risk of diabetes and CVD is similar to or higher than nonvegetarians, which is consistent with findings in western populations [81]. The evidence supporting the health benefits of physical activity is extensive, and it plays a crucial role in preventing both primary and secondary CAD. Epidemiological studies indicate that there is nearly a 50% lower incidence of CAD in physically active individuals compared to those who are sedentary. Being physically inactive is recognized as one of the most significant modifiable risk factors for cardiovascular morbidity and mortality by numerous national and international organizations [82].

There is limited recent national data on sedentary behavior in the middle-aged and elderly population of India. Older people in India have a higher percentage of smokers (26.1% compared) and are more likely to be physically inactive (88.7% compared to 70.7% for vigorous activities, and 67.1% compared to 57.1% for moderate activities when compared to those in better health) [83]. Various local studies have been conducted, such as one in Tamil Nadu in 2010 which found that inadequate physical activity was predominant in 63.3% of the urban population and 40.6% of the rural population [84]. Another study conducted in Madhya Pradesh between 2017 and 2019 found that 19.6% of adults had inadequate physical activity [85]. Similarly, a study conducted among rural adults in South India found that 46.8% of the population was physically inactive [84]. In a national study conducted in 2007–2008 among the older population (≥ 50 years) in India, it was found that 22.0% of them had low physical activity levels [86]. Another study conducted in India in 2007–2008 focused on middle-aged and older adults (aged 50 and above) and found that 22.0% of the selected population had low levels of physical activity [87]. Recently, a study found that nearly 40% of middle-aged and older adults in India had insufficient physical activity levels. The study also identified risk factors for physical inactivity in both men and women. To address this issue, interventions may need to be implemented at multiple levels and should take into account the different physical activity patterns of men and women [88]. According to the findings of ICMR India Diabetes (ICMReINDIAB), half of the individuals surveyed were not engaging in physical activity, and fewer than 10% were participating in consistent exercise routines [3]. Physical inactivity may be associated with the presence of some serious health conditions such as diabetes, CAD, HT, stroke, obstructive pulmonary disease, asthma, depressive symptoms, and sleep problems [89].

### 4.7. Dietary Habits

An unhealthy diet is a major risk factor for CVDs and is linked to other CVD risk factors like high BP, diabetes, and obesity. That is why promoting a healthy diet is crucial in reducing the incidence and mortality rates of CVDs [90]. Diet plays a significant role as a CVRF among older adults in India. Unhealthy dietary habits, characterized by high intake of saturated fats, processed foods, salt, and sugars, contribute to the development of CVDs in India. Additionally, inadequate consumption of fruits, vegetables, and whole grains, which are essential for maintaining heart health, further exacerbates the risk. The prevalence of CVD among older adults in India is influenced by dietary patterns, making dietary interventions an important aspect of preventive strategies aimed at reducing the burden of CVDs in this population.

About half of the Indian population is vegetarian, and yet, CVD risks are comparable with or higher than nonvegetarians as seen in the western population. Indians consume carbohydrate-rich diets along with uneven dietary patterns. Average Indian diets contain more amounts of carbohydrates, high-fat dairy, butter, ghee, and cheese in their everyday meals [3]. In Kerala, the culture and practice of using coconut oil in cooking have predisposed them to the highest rates of CAD in India. Reusing oil for cooking in Indian culture is common, and it increases trans fatty acids leading to a rise in CVDs. Indians consume less amounts of fresh fruits and vegetables compared with the rest of the world. Poor living conditions along with low education levels were also associated with higher CAD mortality in India [91]. Furthermore, the Indian diet consists of refined cereals that lack bran and germ, resulting in a high glycemic index. Protein sources are typically of low quality, primarily derived from legumes which are deficient in the essential amino acid methionine. Gravies used in Indian cuisine are often rich in saturated fats and salt. Fresh fruits, vegetables, and pulses are consumed in relatively low quantities. An increase in sugar consumption (from both traditional sources and from sugar-sweetened beverages) has been recorded in India leading to metabolic disorders including CVDs [92]. Encouraging education and awareness regarding the development of CVDs, discouraging smoking and tobacco consumption, and adopting a nutritious diet and regular exercise regimen can enhance cardiovascular well-being. Decreasing intake of high-fat dairy, carbohydrates, and saturated fats while increasing the consumption of fruits and vegetables can contribute to better overall health. Early initiation of comprehensive screening tests can be advantageous for prompt identification and management. Promoting communal participation in healthy physical activities like walking, yoga, and meditation on a regular basis can significantly contribute to preventing the increasing prevalence of CVDs in India.

## 5. Strategies to Enhance Cardiovascular Prevention and Policies in India

### 5.1. Strategies to Enhance Cardiovascular Prevention

**Figure 4:**
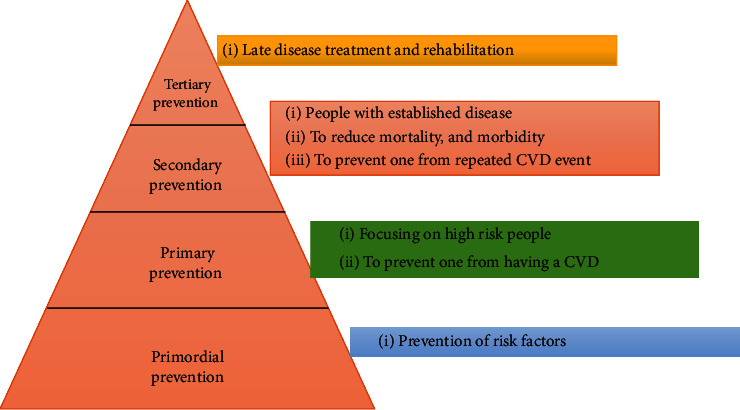
Different levels of prevention of cardiovascular diseases.

High-income countries have witnessed a decline in age-adjusted mortality rates for CVDs in recent decades, primarily due to favorable trends in population-level risk factors such as high cholesterol levels, smoking, and systolic BP. Additionally, improvements in secondary prevention and acute care have also played a role in reducing mortality rates [93]. India will also need to implement primordial, primary, secondary, and tertiary prevention strategies on a larger scale to decrease the burden of CVDs. However, due to limited resources and the larger population in India, more innovative approaches may be required to address this challenge effectively [94]. The following are the prevention and treatment measures that have been successful in tackling the CVD epidemic in India (Figure 4).

#### 5.1.1. Primordial Prevention

Primordial prevention focuses on preventing the development of risk factors by promoting healthy lifestyles that maintain optimal levels of cholesterol, BP, and body weight, and avoiding tobacco use. This approach aims to prevent the need for medical interventions or procedures to treat risk factors and diseases [95]. Dietary intake plays a crucial role in influencing various CVRFs and is considered a key target for primordial prevention interventions. Excessive dietary salt intake has long been known to raise BP levels, with a more significant impact observed in certain groups such as African Americans. Despite some recent controversies, recommendations regarding dietary sodium intake continue to emphasize its adverse effects on BP [96, 97]. Fruit and vegetable-rich diets have been suggested for cardioprotective effects due to their high potassium content, in addition to their low sodium content [98]. Limiting alcohol intake has been shown to be an effective strategy in reducing the risk of developing high BP. Decreasing excessive alcohol consumption can lead to a reduction in BP levels by up to 4 mmHg [99]. In 2014, the Indian government increased the excise duty on tobacco products by 72%, as demonstrated in the preliminary results of the Global Adult Tobacco Survey-2 India (2016–2017).

#### 5.1.2. Primary Prevention

In India, the prevention of risk factors to cure CVDs is not satisfactory. For example, the utilization of therapeutic approaches supported by evidence for the treatment of HT is considerably low when compared to developed economies like the United States. In both urban and rural regions of India, the percentage of people with HT who receive proper care and treatment is comparatively low. Specifically, in rural areas, only 24.9% receive treatment, while in urban areas, the proportion has increased up to 37.6% [100]. Like HT, diabetes management in India is not at an optimal level, with only 33% of patients with diabetes reporting glycosylated hemoglobin (HBA1c) levels below 7% [101]. In order to prevent CVDs in India, there is a need to introduce new, affordable strategies to modify CVRFs and to ensure that these strategies are implemented, scaled up, and sustained over time. In 2016, an analysis based on a microsimulation model revealed that a benefit-based personalized treatment approach focused on reducing CVD risk was more efficient and cost-effective in decreasing CVD deaths in India compared to a treat-to-target approach (which targets a specific BP level) or a hybrid strategy recommended by the WHO [102].

#### 5.1.3. Secondary Prevention

The utilization of preventive therapies to reduce the risk of recurrent CHD and stroke in India is not optimal. The strategies for enhancing adherence among individuals in the secondary prevention population are similar to those employed in the primary prevention population. These include implementing fixed-dose combination therapy, and utilizing task-sharing interventions integrating interventions with many components [103]. Cardiac rehabilitation is an essential part of a secondary prevention program, and when combined with other strategies, it can lead to significant improvements in various health outcomes such as all-cause mortality, cardiovascular mortality, and rehospitalization rates [104]. According to the latest information available, it seems that in India, patients seeking secondary treatment for the prevention of heart disease at a later stage are comparatively younger and have a greater number of risk factors associated with CVDs. To address this issue, it is necessary for health professionals working in various sectors such as government, private, and academic to collaborate and identify gaps in knowledge. This data can then be used to inform policy-making and implement appropriate measures to tackle the problem [105]. Although cardiac rehabilitation has many benefits, including favorable effects on patients, the referral rate for such programs remains low, even in high-income countries. Additionally, in India, the availability of cardiac rehabilitation programs is almost nonexistent. However, traditional approaches like yoga, which can positively affect physical activity and autonomic function, among other benefits for CVD patients, are more prevalent in India and may be more accepted and utilized due to cultural adaptations [15]. Secondary preventive measures can have several benefits, including reducing healthcare costs, improving economic productivity, and enhancing quality of life. These interventions are highly cost-effective, resulting in savings that are three times higher than the country's GDP per capita [106].

#### 5.1.4. Tertiary Prevention

The reduction in CVD death rate in developed countries over the past few decades can be attributed to the success of primary and secondary prevention methods. Nevertheless, the decline in cardiovascular conditions can also be attributed to the effective implementation of evidence-based management for individuals with acute cardiovascular conditions. Registries of patients who have experienced ACS in India have revealed that the management of ACS in India has not been as advanced as it is in the United States. A report on the 2013 ACS registry in the state of Kerala, which has relatively better health indicators compared to other states in India, identified several areas where the quality of care could be improved [107]. The ACS QUIK trial was developed using data obtained from the Kerala ACS Registry. This trial, which involved 21,374 participants from 63 hospitals in India, used a cluster-randomized, stepped-wedge design to evaluate a quality improvement intervention aimed at improving both process-of-care measures and clinical outcomes. Results showed that the intervention led to improvements in process-of-care measures and a 1.1% reduction in the rate of major adverse cardiovascular events at 30 days in the intervention group compared to the control group (6.4% versus 5.3%) [108]. According to a 2015 modeling study, providing national insurance coverage for primary and secondary prevention, as well as tertiary treatment for CVDs, in India, would be a reasonably cost-effective strategy for reducing the burden of the disease. The study compared this approach to the current status quo of having no coverage and found an incremental cost-effectiveness ratio of $1331 DALY averted. This approach was found to be cost-effective across various levels of treatment access and adherence [109]. Policies such as tobacco taxation, reduction of salt in processed foods, and HT treatment are cost-effective measures that can reduce CVDs in LMICs like India. These policies can also help alleviate poverty.

### 5.2. Health Policies

Since most people in India do not have access to health insurance, most medical costs are paid for out of pocket. In India, individuals from lower socioeconomic backgrounds typically depend on the public healthcare system, which has constrained resources to provide adequate care for both acute and chronic CVDs. According to India's draft National Health Policy 2015, a fundamental goal should be to have a preventive and promotive healthcare orientation in all developmental policies and to have a universal access to high-quality healthcare services [110]. The health policy in India has a goal of encouraging the use of health insurance as the main method of safeguarding against financial risks related to healthcare. It also suggests purchasing secondary and tertiary medical services from public and nonprofit private hospitals. Additionally, the policy supports the establishment of a primary care delivery system that is funded through taxes and provided by both public and nonprofit private sectors [111]. India has launched several health financing schemes, but they demand vigorous assessment. Although private and social health insurance can be effective methods of financing healthcare, they are unlikely to fully compensate for the absence of a well-functioning public health system [112]. In order for India's healthcare system to adequately address chronic conditions such as CVDs and NCDs, the suggested universal health coverage by the central government should encompass comprehensive care for such conditions. Furthermore, it should extend benefits beyond individual health to ensure that families are financially protected.

Several countries, including India, use the WHO Model List of Essential Medicines as a reference for their national medicine list. The WHO first released the Essential Medicines List in 1977, which has since expanded to include more than 400 drugs and has been embraced by over 100 nations [113]. WHO defines the Essential Medicines List as a collection of medications that meet the healthcare requirements of a population and are meant to be accessible at all times at a price that individuals and communities can afford. In 1996, India adopted its own National List of Essential Medicines (NLEM), which has since been expanded to include medical devices and procedures [114]. In 2016, an unprecedented decision was made to add stents to the NLEM, which enabled the government to cap their prices. Previously, stents were subject to significant price markups by intermediate distributors, which could be up to four times their original value. After stents were added to the NLEM, their prices were reduced by up to 85% [115].

India's generic drug manufacturing sector provides a higher level of availability of essential cardiovascular drugs compared to other middle-income countries, even though there may be some significant differences between the lists of essential medicines used by these countries. Although India's generic drug manufacturing sector provides a higher level of availability of essential cardiovascular drugs, access to these medicines also includes affordability, which remains a challenge in India. Despite the lower costs of essential CVD medicines in India, more than 45% of overall out-of-pocket health spending is still being spent on medicines [116]. Just as the EDL has played an important role in improving healthcare delivery by increasing patient access to medications, it is believed that an EDL could similarly improve patient access to high-quality diagnostic services in India [117]. India has decided to incorporate certain laboratory tests that are not presently listed on the WHO EDL, as well as some nonlaboratory diagnostic methods (Table 3).

## 6. Limitations of the Study

The current study offers insightful information about the prevalence of CVDs among older adults in India and offers comprehensive management and prevention strategies; however, there are several limitations. Firstly, the study may rely on existing literature, which could be subject to publication bias, potentially overlooking unpublished data or studies with null findings. Secondly, the generalizability of the findings may be limited due to variations in healthcare access, socioeconomic status, and cultural factors across different regions of India. Additionally, the study's recommendations may not fully account for the complexities of implementing public health interventions in diverse settings, such as rural areas with limited resources. Furthermore, there may be gaps in the data regarding specific risk factors or subpopulations within the older adult demographic, which could impact the accuracy of the proposed interventions. Finally, while the study emphasizes the importance of future research to address these limitations, the feasibility and sustainability of such research initiatives may pose challenges in resource-constrained environments. The gaps in available knowledge on the CVD burden, prevention, and control strategies from India and the research and policy needs should be also addressed. For instance, health system preparedness to implement primordial and primary prevention strategies and management of CVDs in primary and secondary care settings needs to be summarized.

## 7. Conclusion and Prospects

CVD is a significant concern for public health in India, and it commonly affects individuals during the most productive periods of their lives especially older adults. CVDs pose a significant threat to the older adult population in India and have multiple causes and risk factors. A higher prevalence of undiagnosed and uncontrolled heart disease and its risk factors among middle-aged and older Indians manifests alarming public health concerns and demands a healthy future. Identifying and managing these risk factors can significantly reduce the incidence of CVD and enhance the quality of life for older adults in India.

India can significantly tackle CVDs among older adults by implementing comprehensive public health campaigns to raise awareness about risk factors such as unhealthy diet, lack of physical activity, smoking, and excessive alcohol consumption. Access to healthcare services should be improved, particularly in rural areas, by establishing more primary healthcare centers equipped for basic CVD screening and management. Health education programs should be implemented to inform older adults about the importance of regular check-ups, medication adherence, and lifestyle modifications to manage CVD risk factors. Promotion of healthy lifestyles, including regular exercise, healthy eating habits, smoking cessation, and moderation in alcohol consumption, should be encouraged through community programs and partnerships with local organizations. Regular screening programs for CVD risk factors like HT, diabetes, and high cholesterol should be conducted among older adults to enable early detection and intervention.

Further research is needed to understand the unique factors contributing to CVDs in the Indian population and develop targeted interventions to address this growing public health concern. Furthermore, the increasing prevalence of CVDs and its detrimental impact on individuals, families, and entire populations demand immediate action. Novel approaches are necessary to arrest the escalation of the CVD epidemic in India's low-resource settings. Overall, concerted efforts are needed from healthcare professionals, policymakers, and the public to effectively prevent and manage CVDs among older adults in India.

## Figures and Tables

**Table 1:** The population attributable risks expressed as percentages for different cardiovascular risk factors concerning coronary heart disease and stroke in India [41].

**Risk factor**	**Interheart (acute myocardial infarction)**	**Interstroke (thrombotic or hemorrhagic strokes)**
Apolipoprotein A/B ratio	49.2	24.9
Diabetes history	9.9	5.0
Hypertension	17.9	34.6
Regular physical activity	12.2	28.5
Psychosocial stress	32.5	9.8
Diet/diet score	13.7	18.8
High waist–hip ratio	20.1	26.5
Lack of alcohol intake	6.7	3.8

**Table 2:** Prevalence of hypercholesterolemia in different states of India.

**States and site**	**Sample size**	**Urban/rural**	**Year**	**Prevalence %**
Indian Industrial Population Surveillance Study	10,442	Urban	2006	Men: 25.1^a^ Women^b^

India Migration Study	1983	Rural	2010	Men: 25.1 Women: 27.8

ICMR Integrated Disease Surveillance Project	15,223	Rural	2010	Men: 31.7 Women: 32.8

ICMR Integrated Disease Surveillance Project	13,517	Rural	2010	Men: 19.5 Women: 26.4

ICMR Integrated Disease Surveillance Project	15,751	Periurban/urban slum	2010	Men: 18.1 Women: 23.4

Indian Women's Health Study	2008	Urban	2013	Women: 27.7^c^

Indian Women's Health	2616	Rural	2013	Women: 13.5^c^

India Heart Watch	6123	Urban	2014	Men: 25.1 Women: 24

ICMR-INDIAB Study	2042	Rural and urban	2014	Men: 13.9^a^ Women^b^

Fit Heart Study	46919	Urban	2014	Men: 29.0 Women: 30.8

Aggarwal community	2500	Rural	2015	Men: 25.6 Women^b^

Punjab, India	5127	Rural and urban	2017	Men: 9.8 Women: 9.8

ICMR-INDIAB Study: Rural & Urban	18,492	Rural and urban	2023	Men: 23.2 Women: 24.8

^a^Prevalence for men and women combined.

^b^Sex-specific data not available.

^c^Only women participated in this study.

**Table 3:** Key diagnostic tests for cardiovascular disease in India.

**India draft EDL**	**Present on WHO EDL first edition**	**Comments**
*Subhealth centers*		
Hemoglobin	Yes	Tests related to CVDs and their risk factors, such as glucose and urinary albumin tests, should be accessible at facilities that do not have proper laboratory equipment.

*Primary health centers*		
Creatinine	Yes	Except for CXR and ECG, most of the tests are suggested in both India's proposed EDL and WHO EDL. However, TSH is not currently included in the WHO EDL. These laboratory tests can be conducted using semiautomated analyzers and are appropriate for facilities with limited infrastructure and low testing volumes.
Electrolytes	Yes	
Liver function tests	Yes	
Lipid profile	Yes	
CXR	NA	
Electrocardiograms	NA	
Thyroid-stimulating hormone	No	

*Community health centers*		
Blood culture (by specimen transport to district level)	Yes	The diagnosis of endocarditis can be aided by performing blood cultures.

*District health centers*		
PT	Yes	India's EDL includes all of these tests, but they are only recommended to be conducted at the district level. However, we suggest that these tests should be accessible at lower tiers of India's healthcare system. The expenses associated with providing automated or semiautomated immunoassay testing may pose a limitation.
PTT	Yes	
INR	Yes	
Troponin I/T	Yes	
CK-MB	No	
Arterial blood gas with lactic acid	Yes	Blood gas testing may be necessary at community health centers that have the capability to perform surgical procedures.
MRI	NA	Not all district health centers provide advanced medical care. Some diagnostic tests, such as MRI, may only be obtainable at tertiary hospitals.
Coronary and peripheral angiography	NA	

*Supplementary diagnostic tests excluded from India's Essential Diagnostic List*		
B-natriuretic peptide		A potential barrier is cost.
Thiocyanate level		These diagnostic tests would only be conducted at district health centers that have specialized medical services.
Noninvasive stress testing		It is recommended to include these diagnostic tests as they can help rule CAD in populations with low to intermediate risk.
CT		CT should be provided to aid in the diagnosis of CAD, PAD, and acute aortic syndromes.

Abbreviations: CAD, coronary artery disease; CK-MB, creatinine-kinase; CT, computed tomography; CXR, chest X-ray; ECH, electrocardiogram; EDL, Essential Diagnostics List; INR, international normalized ratio; MRI, magnetic resonance imaging; PAD, peripheral artery disease; PAI-1, plasminogen activator inhibitor; PT, prothrombin time; PTT, partial thromboplastin time; TSH, thyroid-stimulating hormone.

## Data Availability

The datasets are contained within this manuscript.

## Nomenclature

ACS: Acute coronary syndrome
BP: Blood pressure
CAD: Coronary artery disease
CHD: Coronary heart disease
CK-MB: Creatinine-kinase
CRDs: Chronic respiratory diseases
CT: Computed tomography
CXR: Chest X-ray
DALYs: Disability-adjusted life years
EAG: Empowered action group
ECG: Electrocardiogram
EDL: Essential diagnostic list
GDP: Gross domestic product
HDL: High-density lipoprotein
HT: Hypertension
ICMReINDIAB: Indian Council of Medical Research India Diabetes
ICMR-INDIAB: Indian Council of Medical Research-India Diabetes
INR: International normalized ratio
JHW: Jaipur Heart Watch
LASI: Longitudinal ageing study in India
LDL: Low-density lipoprotein
LMICs: Low- and middle-income countries
MRI: Magnetic resonance imaging
NLEM: National List of Essential Medicines
PAI-1: Plasminogen activator inhibitor
PT: Prothrombin time
PTT: Partial thromboplastin time
SEP: Socioeconomic positions
SES: Socioeconomic status
TG: Triglycerides
TSH: Thyroid-stimulating hormone
VLDL: Very low-density lipoprotein
WHO: World Health Organization

## References

[1] Thomas H., Diamond J., Vieco A. (2018). Global atlas of cardiovascular disease 2000-2016: the path to prevention and control. *Global Heart*.

[2] Joshi P., Islam S., Pais P. (2007). Risk factors for early myocardial infarction in South Asians compared with individuals in other countries. *Jama*.

[3] Kumar A. S., Sinha N. (2020). Cardiovascular disease in India: a 360 degree overview. *Medical Journal Armed Forces India*.

[4] Kalra A., Jose A. P., Prabhakaran P. (2023). The burgeoning cardiovascular disease epidemic in Indians–perspectives on contextual factors and potential solutions. *The Lancet Regional Health-Southeast Asia*.

[5] Li Z., Lin L., Wu H. (2021). Global, regional, and national death, and disability-adjusted life-years (DALYs) for cardiovascular disease in 2017 and trends and risk analysis from 1990 to 2017 using the Global Burden of Disease Study and implications for prevention. *Frontiers in Public Health*.

[6] Shi D., Tao Y., Wei L. (2024). The burden of cardiovascular diseases attributed to diet high in sugar-sweetened beverages in 204 Countries and territories from 1990 to 2019. *Current Problems in Cardiology*.

[7] Kundu J., Kundu S. (2022). Cardiovascular disease (CVD) and its associated risk factors among older adults in India: evidence from LASI Wave 1. *Clinical Epidemiology and Global Health*.

[8] Dilnawaz F., Iqbal Z. (2021). *Nanomedicinal Approaches Towards Cardiovascular Disease*.

[9] Wongsawat S. (2017). Predicting factors for quality of life of elderly in the rural areas. *International Journal of Arts and Sciences*.

[10] Miranda J. J., Barrientos-Gutierrez T., Corvalan C. (2019). Understanding the rise of cardiometabolic diseases in low-and middle-income countries. *Nature Medicine*.

[11] Ivanovs R., Rancāns E., Ķīvīte A. (2017). Anxiety associated with a reduction of cardiovascular mortality risk in primary care population in Latvia. *European Neuropsychopharmacology*.

[12] Velayutham B., Kangusamy B., Joshua V., Mehendale S. (2016). The prevalence of disability in elderly in India–analysis of 2011 census data. *Disability and Health Journal*.

[13] Curtis A. B., Karki R., Hattoum A., Sharma U. C. (2018). Arrhythmias in patients ≥80 years of age: pathophysiology, management, and outcomes. *Journal of the American College of Cardiology*.

[14] Kastor A., Mohanty S. K. (2016). Associated covariates of functional limitation among older adults in India: an exploration. *Ageing International*.

[15] Roth G. A., Mensah G. A., Johnson C. O. (2020). Global Burden of Cardiovascular Diseases Writing Group. Global Burden of Cardiovascular Diseases and Risk Factors, 1990-2019: Update from the GBD 2019 study. *Journal of the American College of Cardiology*.

[16] Arokiasamy P., Agrawal G. (2010). Population attributable risk fraction for selected chronic diseases in India. *Journal of Primary Care and Community Health*.

[17] Yusuf S., Rangarajan S., Teo K. (2014). Cardiovascular risk and events in 17 low-, middle-, and high-income countries. *The New England Journal of Medicine*.

[18] Ahmed W., Muhammad T., Maurya C., Akhtar S. N. (2023). Prevalence and factors associated with undiagnosed and uncontrolled heart disease: a study based on self-reported chronic heart disease and symptom-based angina pectoris among middle-aged and older Indian adults. *PLoS One*.

[19] Mullins A., Akhavan N., Arjmandi B., Ormsbee L. (2022). Study protocol: effects of daily prune consumption on lipid profile, inflammation, and oxidative stress. *Current Developments in Nutrition*.

[20] Abdul-Aziz A. A., Desikan P., Prabhakaran D., Schroeder L. F. (2019). Tackling the burden of cardiovascular diseases in India. *Circulation Cardiovascular Quality and Outcomes*.

[21] Prabhakaran D., Jeemon P., Roy A. (2016). Cardiovascular diseases in India. *Circulation*.

[22] Shah H., Altaf A., Salahuddin M., Jan M. U., Khan A. (2018). Cardiovascular risk factors of hypertension, smoking and obesity: emerging concerns among Pathan and Persian young adults?. *Medical Journal of the Islamic Republic of Iran*.

[23] Zhao D. (2021). Epidemiological features of cardiovascular disease in Asia. *JACC: Asia*.

[24] Chauhan S., Aeri B. T. (2013). Prevalence of cardiovascular disease in India and it is economic impact-a review. *International Journal of Scientific and Research Publications*.

[25] Ralapanawa U., Sivakanesan R. (2021). Epidemiology and the magnitude of coronary artery disease and acute coronary syndrome: a narrative review. *Journal of Epidemiology and Global Health*.

[26] Thrift A. G., Ragavan R. S., Riddell M. A. (2020). Hypertension in rural India: the contribution of socioeconomic position. *Journal of the American Heart Association*.

[27] Akhtar S. N., Saikia N. (2023). Economic dependency, chronic illness, and insurance coverage among the elderly. *Handbook of Aging, Health and Public Policy*.

[28] Mishra A., Mokashi T., Nair A., Chokshi M. (2022). Mapping healthcare data sources in India. *Journal of Health Management*.

[29] Shah B., Mathur P. (2010). Surveillance of cardiovascular disease risk factors in India: the need & scope. *The Indian Journal of Medical Research*.

[30] John R. M., Dauchy E. P. (2022). Healthcare costs attributable to secondhand smoke exposure among Indian adults. *Nicotine & Tobacco Research*.

[31] Prabhakaran D., Jeemon P., Sharma M. (2018). The changing patterns of cardiovascular diseases and their risk factors in the states of India: the Global Burden of Disease Study 1990–2016. *The Lancet Global Health*.

[32] Kalra A., Jose A. P., Prabhakaran P. (2023). The burgeoning cardiovascular disease epidemic in Indians - perspectives on contextual factors and potential solutions. *The Lancet Regional Health - Southeast Asia*.

[33] Ruan Y., Guo Y., Zheng Y. (2018). Cardiovascular disease (CVD) and associated risk factors among older adults in six low-and middle-income countries: results from SAGE Wave 1. *BMC Public Health*.

[34] Gupta R., Mohan I., Narula J. (2018). Trends in coronary heart disease epidemiology in India. *Annals of Global Health*.

[35] Banerjee S., Kumar P., Srivastava S., Banerjee A. (2021). Association of anthropometric measures of obesity and physical activity with cardio-vascular diseases among older adults: evidence from a cross-sectional survey, 2017–18. *PLoS One*.

[36] Gupta R., Joshi P., Mohan V., Reddy K. S., Yusuf S. (2008). Epidemiology and causation of coronary heart disease and stroke in India. *Heart*.

[37] Jana A., Chattopadhyay A. (2022). Prevalence and potential determinants of chronic disease among elderly in India: rural-urban perspectives. *PLoS One*.

[38] Chauhan S., Aeri B. T. (2015). The rising incidence of cardiovascular diseases in India: assessing its economic impact. *European Journal of Preventive Cardiology*.

[39] Sekhri T., Kanwar R. S., Wilfred R. (2014). Prevalence of risk factors for coronary artery disease in an urban Indian population. *BMJ Open*.

[40] Sutradhar S. R. (2020). The impact of remittances on economic growth in Bangladesh, India, Pakistan and Sri Lanka. *International Journal of Economic Policy Studies*.

[41] Gupta R., Deedwania P. C., Sharma K. (2012). Association of educational, occupational and socioeconomic status with cardiovascular risk factors in Asian Indians: a cross-sectional study. *PLoS One*.

[42] Wu C.-Y., Hu H.-Y., Chou Y.-J., Huang N., Chou Y.-C., Li C.-P. (2015). High Blood Pressure and All-Cause and Cardiovascular Disease Mortalities in Community-Dwelling Older Adults. *Medicine*.

[43] Zakir F., Mohapatra S., Farooq U., Mirza M. A., Iqbal Z. (2022). Introduction to metabolic disorders. *Drug Delivery Systems for Metabolic Disorders*.

[44] Carson A. P., Howard G., Burke G. L., Shea S., Levitan E. B., Muntner P. (2011). Ethnic differences in hypertension incidence among middle-aged and older adults. *Hypertension*.

[45] Egan B. M., Zhao Y., Axon R. N. (2010). US trends in prevalence, awareness, treatment, and control of hypertension, 1988-2008. *JAMA*.

[46] Singh R. B., Suh I. L., Singh V. P. (2000). Hypertension and stroke in Asia: prevalence, control and strategies in developing countries for prevention. *Journal of Human Hypertension*.

[47] Anchala R., Kannuri N. K., Pant H. (2014). Hypertension in India: a systematic review and meta-analysis of prevalence, awareness, and control of hypertension. *Journal of Hypertension*.

[48] Bhadoria A. S., Kasar P. K., Toppo N. A., Bhadoria P., Pradhan S., Kabirpanthi V. (2014). Prevalence of hypertension and associated cardiovascular risk factors in Central India. *Journal of Family and Community Medicine*.

[49] Ramakrishnan S., Zachariah G., Gupta K. (2019). Prevalence of hypertension among Indian adults: results from the great India blood pressure survey. *Indian Heart Journal*.

[50] Bhatia M., Kumar M., Dixit P., Dwivedi L. K. (2021). Diagnosis and treatment of hypertension among people aged 45 years and over in India: a sub-national analysis of the variation in performance of Indian states. *Frontiers in Public Health*.

[51] Farron M. R., Kabeto M. U., Dey A. B., Banerjee J., Levine D. A., Langa K. M. (2020). Hypertension and cognitive health among older adults in India. *Journal of the American Geriatrics Society*.

[52] Banerjee A., Chen S., Pasea L. (2021). Excess deaths in people with cardiovascular diseases during the COVID-19 pandemic. *European Journal of Preventive Cardiology*.

[53] Tsukru V. (2023). Prevalence and socio-demographic determinants of obesity and hypertension in a rural tribal community in northeast india. *Online Journal of Health and Allied Sciences*.

[54] Rengma M. S., Sen J., Mondal N. (2015). Socio-economic, demographic and lifestyle determinants of overweight and obesity among adults of Northeast India. *Ethiopian Journal of Health Sciences*.

[55] Ahirwar R., Mondal P. R. (2020). The prevalence of hypertension in Indian populations in last decade. *Asian Man (The)-An International Journal*.

[56] Bramhankar M., Pandey M., Rana G. S., Rai B., Mishra N. L., Shukla A. (2021). An assessment of anthropometric indices and its association with NCDs among the older adults of India: evidence from LASI Wave-1. *BMC Public Health*.

[57] Gupta R., Rao R. S., Misra A., Sharma S. K. (2017). Recent trends in epidemiology of dyslipidemias in India. *Indian Heart Journal*.

[58] Enas E. A., Dharmarajan T. S., Varkey B. (2015). Consensus statement on the management of dyslipidemia in Indian subjects: A different perspective. *Indian Heart Journal*.

[59] Guptha S., Gupta R., Deedwania P. (2014). Cholesterol lipoproteins and prevalence of dyslipidemias in urban Asian Indians: a cross sectional study. *Indian Heart Journal*.

[60] Joshi S. R., Anjana R. M., Deepa M. (2014). Prevalence of dyslipidemia in urban and rural India: the ICMR–INDIAB study. *PLoS One*.

[61] Tripathy J. P., Thakur J. S., Jeet G. (2017). Burden and risk factors of dyslipidemia-results from a STEPS survey in Punjab India. *Diabetes and Metabolic Syndrome: Clinical Research & Reviews*.

[62] Erhardt L. (2009). Cigarette smoking: an undertreated risk factor for cardiovascular disease. *Atherosclerosis*.

[63] Niaz K., Maqbool F., Khan F., Bahadar H., Hassan F. I., Abdollahi M. (2017). Smokeless tobacco (Paan and Gutkha) consumption, prevalence, and contribution to oral cancer. *Epidemiology and Health*.

[64] Sathish T., Kannan S., Sarma P. S., Thankappan K. R. (2015). Incidence of tobacco use among adults (15-64 years) in rural Kerala. *Asia Pacific Journal of Public Health*.

[65] Puig-Cotado F., Tursan d’Espaignet E., St Claire S. (2020). *Tobacco and Coronary Heart Disease: WHO Tobacco Knowledge Summaries*.

[66] John R. M., Sinha P., Munish V. G., Tullu F. T. (2021). Economic costs of diseases and deaths attributable to tobacco use in India, 2017–2018. *Nicotine and Tobacco Research*.

[67] Csordas A., Bernhard D. (2013). The biology behind the atherothrombotic effects of cigarette smoke. *Nature Reviews Cardiology*.

[68] Barua R. S., Ambrose J. A. (2013). Mechanisms of coronary thrombosis in cigarette smoke exposure. *Arteriosclerosis, Thrombosis, and Vascular Biology*.

[69] Basu S., Glantz S., Bitton A., Millett C. (2013). The effect of tobacco control measures during a period of rising cardiovascular disease risk in India: a mathematical model of myocardial infarction and stroke. *PLoS Medicine*.

[70] Kumar A., Siddharth V., Singh S. I., Narang R. (2022). Cost analysis of treating cardiovascular diseases in a super-specialty hospital. *PLoS One*.

[71] Adasuriya G., Haldar S. (2023). Next generation ECG: the impact of artificial intelligence and machine learning. *Current Cardiovascular Risk Reports*.

[72] Ruhil R. (2018). India has reached on the descending limb of tobacco epidemic. *Indian Journal of Community Medicine*.

[73] Das U., Kar N. (2023). Prevalence and risk factor of diabetes among the elderly people in West Bengal: evidence-based LASI 1st wave. *BMC Endocrine Disorders*.

[74] Shetty D., Dua M., Kumar K., Dhanpal R., Astekar M., Shetty D. C. (2012). Oral hygiene status of individuals with cardiovascular diseases and associated risk factors. *Clinics and Practice*.

[75] Mohan V., Venkatraman J. V., Pradeepa R. (2010). Epidemiology of cardiovascular disease in type 2 diabetes: the Indian scenario. *Journal of Diabetes Science and Technology*.

[76] Einarson T. R., Acs A., Ludwig C., Panton U. H. (2018). Prevalence of cardiovascular disease in type 2 diabetes: a systematic literature review of scientific evidence from across the world in 2007–2017. *Cardiovascular Diabetology*.

[77] Karuranga S., Fernandes J., Huang Y., Malanda B. (2017). *IDF Diabetes Atlas*.

[78] Deedwania P. C. (2014). Statins in chronic kidney disease: cardiovascular risk and kidney function. *Postgraduate Medicine*.

[79] Eitel I., Stiermaier T., Lange T. (2018). Cardiac magnetic resonance myocardial feature tracking for optimized prediction of cardiovascular events following myocardial infarction. *JACC: Cardiovascular Imaging*.

[80] Leon B. M., Maddox T. M. (2015). Diabetes and cardiovascular disease: epidemiology, biological mechanisms, treatment recommendations and future research. *World Journal of Diabetes*.

[81] Shridhar K., Dhillon P. K., Bowen L. (2014). The association between a vegetarian diet and cardiovascular disease (CVD) risk factors in India: the Indian Migration Study. *PLoS One*.

[82] Prasad D. S., Das B. C. (2009). Physical inactivity: a cardiovascular risk factor.

[83] Cramm J. M., Lee J. (2014). Smoking, physical activity and healthy aging in India. *BMC Public Health*.

[84] Devamani C. S., Oommen A. M., Mini G. K., Abraham V. J., George K. (2019). Levels of physical inactivity in rural and urban Tamil Nadu, India: a cross-sectional study. *Journal of Clinical and Preventive Cardiology*.

[85] Kokane A. M., Joshi R., Kotnis A. (2020). Determinants of behavioural and biological risk factors for cardiovascular diseases from state level STEPS survey (2017–19) in Madhya Pradesh. *PeerJ*.

[86] Sarveswaran G., Kulothungan V., Mathur P. (2020). Clustering of noncommunicable disease risk factors among adults (18–69 years) in rural population, South-India. *Diabetes and Metabolic Syndrome: Clinical Research & Reviews*.

[87] Koyanagi A., Stubbs B., Smith L., Gardner B., Vancampfort D. (2017). Correlates of physical activity among community-dwelling adults aged 50 or over in six low- and middle-income countries. *PLoS One*.

[88] Pengpid S., Peltzer K. (2022). Prevalence and associated factors of physical inactivity among middle-aged and older adults in India: results of a national cross-sectional community survey. *BMJ Open*.

[89] De Ramirez S. S., Enquobahrie D. A., Nyadzi G. (2010). Prevalence and correlates of hypertension: a cross-sectional study among rural populations in sub-Saharan Africa. *Journal of Human Hypertension*.

[90] O’Donnell M. J., Chin S. L., Rangarajan S. (2016). Global and regional effects of potentially modifiable risk factors associated with acute stroke in 32 countries (INTERSTROKE): a case-control study. *Lancet*.

[91] Do R., Willer C. J., Schmidt E. M. (2013). Common variants associated with plasma triglycerides and risk for coronary artery disease. *Nature Genetics*.

[92] Gulati G., Zhang K. W., Scherrer-Crosbie M., Ky B. (2014). Cancer and cardiovascular disease: the use of novel echocardiography measures to predict subsequent cardiotoxicity in breast cancer treated with anthracyclines and trastuzumab. *Current Heart Failure Reports*.

[93] Allender S., Lacey B., Webster P. (2010). Level of urbanization and noncommunicable disease risk factors in Tamil Nadu, India. *Bulletin of the world Health Organization*.

[94] Natalia Swiatoniowska N., Bartosiak E., Szymanska-Chabowska A., Mazur G., Jankowska-Polanska B. (2019). Knowledge and Awareness of Cardio-Vascular Risk Factors as the Predictor of Therapeutical Adherence to Antihypertensive Treatment. *European Journal of Preventive Cardiology*.

[95] Claas S. A., Arnett D. K. (2016). The role of healthy lifestyle in the primordial prevention of cardiovascular disease. *Current Cardiology Reports*.

[96] Mozaffarian D., Fahimi S., Singh G. M. (2014). Global sodium consumption and death from cardiovascular causes. *The New England Journal of Medicine*.

[97] Alderman M. H., Cohen H. W. (2012). Dietary sodium intake and cardiovascular mortality: controversy resolved?. *Current Hypertension Reports*.

[98] Aaron K. J., Sanders P. W. (2013). Role of dietary salt and potassium intake in cardiovascular health and disease: a review of the evidence. *Mayo Clinic Proceedings*.

[99] Kodavali L., Townsend R. R. (2006). Alcohol and its relationship to blood pressure. *Current Hypertension Reports*.

[100] Roy A., Praveen P. A., Amarchand R. (2017). Changes in hypertension prevalence, awareness, treatment and control rates over 20 years in National Capital Region of India: results from a repeat cross-sectional study. *BMJ Open*.

[101] Deepa M., Grace M., Binukumar B. (2015). High burden of prediabetes and diabetes in three large cities in South Asia: the center for cardio-metabolic risk reduction in South Asia (CARRS) study. *Diabetes Research and Clinical Practice*.

[102] Basu S., Yudkin J. S., Sussman J. B., Millett C., Hayward R. A. (2016). Alternative strategies to achieve cardiovascular mortality goals in China and India: a microsimulation of target-versus risk-based blood pressure treatment. *Circulation*.

[103] Yusuf S., Islam S., Chow C. K. (2011). Use of secondary prevention drugs for cardiovascular disease in the community in high-income, middle-income, and low-income countries (the PURE study): a prospective epidemiological survey. *Lancet*.

[104] Heran B. S., Chen J. M. H., Ebrahim S. (2011). Exercise-based cardiac rehabilitation for coronary heart disease. *Cochrane Database of Systematic Reviews*.

[105] Rehman H., Kalra A., Kochar A. (2020). Secondary prevention of cardiovascular diseases in India: findings from registries and large cohorts. *Indian Heart Journal*.

[106] Jankowski P., Czarnecka D., Badacz L. (2018). Practice setting and secondary prevention of coronary artery disease. *Archives of Medical Science*.

[107] Mohanan P. P., Mathew R., Harikrishnan S. (2013). Presentation, management, and outcomes of 25 748 acute coronary syndrome admissions in Kerala, India: results from the Kerala ACS Registry. *European Heart Journal*.

[108] Huffman M. D., Mohanan P. P., Devarajan R. (2018). Effect of a quality improvement intervention on clinical outcomes in patients in India with acute myocardial infarction: the ACS QUIK randomized clinical trial. *JAMA*.

[109] Basu S., Bendavid E., Sood N. (2015). Health and economic implications of national treatment coverage for cardiovascular disease in India: cost-effectiveness analysis. *Circulation. Cardiovascular Quality and Outcomes*.

[110] Bhaumik S. (2012). India outlines plans for national urban health mission. *Lancet*.

[111] Ghosh S. (2011). Catastrophic payments and impoverishment due to out-of-pocket health spending. *Economic and Political Weekly*.

[112] Rao M., Ramachandra S. S., Bandyopadhyay S. (2011). Addressing healthcare needs of people living below the poverty line: a rapid assessment of the Andhra Pradesh health insurance scheme. *National Medical Journal of India*.

[113] Wirtz V. J., Hogerzeil H. V., Gray A. L. (2017). Essential medicines for universal health coverage. *Lancet*.

[114] Manikandan S. (2023). The National list of essential medicines of India 2022 (NLEM 2022): Tommy, Toe the Line. *Lancet Regional Health Southeast Asia*.

[115] Wadhera P., Alexander T., Nallamothu B. K. (2017). India and the coronary stent market. *Circulation*.

[116] Newman C., Ajay V. S., Srinivas R., Bhalla S., Prabhakaran D., Banerjee A. (2016). Drugs for cardiovascular disease in India: perspectives of pharmaceutical executives and government officials on access and development-a qualitative analysis. *Journal of Pharmaceutical Policy and Practice*.

[117] Schroeder L. F., Guarner J., Amukele T. K. (2018). Essential diagnostics for the use of World Health Organization essential medicines. *Clinical Chemistry*.

